# Association of Electronic Cigarette Usage with the Subsequent Initiation of Combustible Cigarette Smoking among Dental Students in Riyadh, Saudi Arabia: A Longitudinal Study

**DOI:** 10.3390/healthcare12111092

**Published:** 2024-05-26

**Authors:** Sanjeev B. Khanagar, Ibrahim Aldawas, Salman Khalid Alrusaini, Farraj Albalawi, Aram Alshehri, Mohammed Awawdeh, Kiran Iyer, Darshan Devang Divakar

**Affiliations:** 1 Preventive Dental Science Department, College of Dentistry, King Saud bin Abdulaziz University for Health Sciences, Riyadh 11426, Saudi Arabia; 2 King Abdullah International Medical Research Centre, Ministry of National Guard Health Affairs, Riyadh 11481, Saudi Arabia; 3 College of Public Health, Texila American University, Georgetown 413741, Guyana; 4 College of Dentistry, King Saud bin Abdulaziz University for Health Sciences, Riyadh 11426, Saudi Arabia; 5 Restorative and Prosthetic Dental Sciences Department, College of Dentistry, King Saud bin Abdulaziz University for Health Sciences, Riyadh 11426, Saudi Arabia; 6 Dental Biomaterials Research Chair, Dental Health Department, College of Applied Medical Sciences, King Saud University, Riyadh 11433, Saudi Arabia; ddivakar@ksu.edu.in

**Keywords:** addiction, combustible cigarette, dental students, dependence, e-cigarettes, initiation, risk factor, nicotine, smoking

## Abstract

The use of electronic cigarettes, or “e-cigarettes”, among youths has sparked worries about the possibility of nicotine dependence as a serious public health issue. Dental practitioners play a critical role in helping their patients quit smoking. Dental schools across the globe have policies encouraging their students to help patients quit smoking. Current research, however, indicates that a significant portion of dental students smoke combustible cigarettes and use e-cigarettes. According to studies, using e-cigarettes has resulted in the subsequent initiation of combustible cigarette smoking among its users. The aim of this study was to determine the association between the use of electronic cigarettes and the subsequent initiation of combustible cigarette smoking among dental students who were not attitudinally susceptible to smoking combustible cigarettes. A longitudinal cohort study was conducted among 121 study participants who were never combustible cigarette users and were attitudinally non-susceptible to smoking at baseline. At baseline, 66 (54.6%) study participants were categorized as e-cigarette users who were attitudinally non-susceptible to combustible cigarette smoking, and 55 (45.4%) study participants were categorized as non-users who were attitudinally non-susceptible to combustible cigarette smoking. The initiation of combustible cigarette smoking was assessed at 6- and 12-month intervals. Binomial regression analysis of the outcome at the end of one-year follow-up, when analyzed with independent variables, revealed a significant influence of e-cigarette use on taking up combustible cigarette smoking [Relative Risk: 9.395; 95% CI: 3.03–29.04]. Chi-squared analysis of independent variables revealed e-cigarette use to be significantly associated with fathers’ education level (*p* = 0.00), parental cigarette smoking status (*p* = 0.00), cigarette smoking among friends (*p* = 0.00), and family income (*p* = 0.00). E-cigarette users are more likely to believe it to be healthier (*p* = 0.00) than combustible smoking. In the present study, e-cigarette usage demonstrated a significant influence on taking up combustible cigarette smoking among its users. Educational institutions should implement stringent policies and regulations to prevent health professionals from using these products.

## 1. Introduction

Electronic cigarettes (e-cigarettes) were developed in the year 1965 and eventually received a patent as a smokeless non-tobacco cigarette (Patent No. 3200.819) [1]. This device was then marketed as a safe and harmless alternative to smoking, with the intent to replace the regular combustible cigarette. Later, e-cigarettes were further developed in China and introduced to the Chinese market [2]. They gained attention among cigarette users in China and in the United States as a product with the potential use for smoking cessation or as an alternative for combustible cigarettes [3,4]. Since then, production companies have extensively promoted these products through television commercials, print advertisements starring celebrities, and internet marketing using social media, which has led to a noticeably large increase in e-cigarette usage among both adults and youths [5,6].

The popularity of these products among youths has raised concerns about the potential for nicotine dependence as a major public health concern, since the Center for Disease Control and Prevention has clearly reported that these products have some potential benefits for some and may pose a harm to others [7]. General health concerns are due to the harmful substances that are released from e-cigarettes. The aerosols released from these products comprise propylene glycol, glycerin, and toxic metals like lead, nickel, cadmium, and carcinogenic carbonyl compounds, which include formaldehyde [8,9,10]. Reports suggest that these harmful substances released from e-cigarettes have the potential to damage the deoxyribonucleic acid (DNA) and can reduce its repairing ability, which may further lead to respiratory diseases [11]. Chronic exposure to e-cigarette vapor can significantly alter the physiology of lung epithelial cells and immune cells, thereby reducing the defensive response to infectious challenges [12]. Long-term exposure to nicotine among diabetic patients has shown resistance to insulin and an increased risk of developing cardiovascular diseases [13,14]. Exposure to nicotine during pregnancy is associated with congenital malformations like cleft lip and palate [15].

E-cigarettes also pose a potential threat to oral health, as they cause increased oxidative and carbonyl stress and the release of inflammatory cytokines in the fibroblasts of the periodontal ligament [16]. Flavored e-cigarettes have the potential to induce significant levels of oxidative stress, cytotoxicity, and genotoxicity in oral epithelial cell lines [17]. A systematic review reported that stomatitis, hairy tongue, xerostomia, and angular cheilitis were more prevalent among e-cigarette users in comparison with combustible cigarette users and non-users [18].

E-cigarette usage is promoted and marketed as a method to reduce nicotine dependence and as an alternative to conventional cigarette smoking [19]. However, the use of e-cigarettes is considered to increase the risk of addiction among youths [20,21]. Considering the health and safety of these products among youths, their sale and marketing are forbidden or banned in several countries, like India, Australia, Brazil, Singapore, Uruguay, and Argentina [22,23,24]. Based on the advice of the Saudi Food and Drug Authority, the Saudi Arabian government banned the import and sale of electronic cigarettes in 2014 [25]. Travelers are now permitted to bring one e-cigarette with a certain quantity of flavorings since the restriction was lifted in 2019. Additionally, entrepreneurs are allowed, by law, to import and market these goods in Saudi Arabia [26].

The aggressive marketing techniques utilized by e-cigarettes companies have generated enormous sales and made these goods easily accessible to their clients, which has led to a significant increase in the prevalence of e-cigarette usage among Saudi Arabia’s adolescents and adults. According to Aqeeli A.A. et al. [25], 21% of Saudi Arabia’s undergraduate students in the Jazan region use e-cigarettes. Another study by Althobaiti N.K et al. [27] found that young adults from the Mecca Region of Saudi Arabia, aged 18 to 24 years old, had a prevalence of 26%. According to a systematic review by Patil S et al. [28], between 10.6% and 27.7% of Saudi Arabian medical students used e-cigarettes. According to Sharanesha RB et al. [29], dental students in the Riyadh region of Saudi Arabia use e-cigarettes at a rate of 21%. A global study by Alhajj MN et al. [30] examined the use of e-cigarettes by dental students in 11 different countries. The results showed that the prevalence of e-cigarette use varied from 1.6% in Lebanon to 11.7% in Saudi Arabia.

Dental practitioners play a critical role in helping their patients quit smoking. Dentists are typically the first to observe the damaging effects of nicotine and tobacco on oral tissue [31]. There are regulations in place at dental schools all over the world that motivate dental students to help patients quit smoking [32]. Additionally, research has shown that patients who receive motivation from their dentists at dental clinics are more likely to give up smoking [33]. Research has also documented differing levels of efficacy among tobacco cessation strategies designed and executed in dental care environments [34,35]. Given the critical role that dentists play in helping people quit smoking, it is imperative that they set a high standard for behavior within their communities. However, the observed common use of e-cigarettes among dental students is highly concerning. In the e-cigarette literature, numerous studies have demonstrated that the use of e-cigarettes by non-users of combustible cigarettes had led to the eventual initiation of combustible cigarettes smoking [36,37,38,39,40,41,42,43,44,45,46,47,48,49]. The results of these studies have issued a warning, stating that electronic cigarettes, which are marketed as a tobacco cessation aid or an alternative to combustible cigarettes, can actually act as a product that encourages the smoking of combustible cigarettes.

This study was conducted in Riyadh, Saudi Arabia, with the intention of determining the association between electronic cigarette usage with the subsequent initiation of combustible cigarette smoking among dental students who were attitudinally non-susceptible to smoking combustible cigarettes. The study’s objectives were to ascertain the impact of independent variables, such as the gender of the participants, the educational attainment of their parents, the smoking habits of their parents, and the smoking habits of their friends, on the research findings.

## 2. Materials and Methods

### 2.1. Research Design

A longitudinal cohort study design was adopted for this research, which was scheduled over a period of twelve months.

A research protocol was submitted to the Institutional Review Board of the King Abdullah International Medical Research Center (KAIMRC), located in Riyadh, Saudi Arabia, prior to the commencement of data collection. Ethical clearance was acquired, and the research protocol was authorized (Ref. No. IRBC/2374/21 Study Number: NRC21R/469/10).

### 2.2. Sample Size Estimation

In our study, a power analysis was conducted to determine the necessary sample size for achieving adequate statistical power. Based on an estimated proportion of 0.023 in group I and 0.189 in group II, we expected to observe a risk difference of −0.166. Employing a two-sided test with an alpha level of 5%, and aiming for a power of 90%, the required sample size for each group was calculated to be 57 participants, which is a total of 114 participants using the sample size estimation formula [50].

### 2.3. Sampling Technique

A non-probability sampling technique was adopted for this study design. The convenience sampling technique [snowball technique] was adopted for recruiting the study subjects.

### 2.4. Eligibility Criteria

#### 2.4.1. Inclusion Criteria

Dental students who signed a written informed consent form and were willing to participate, participants who were never cigarette users and were attitudinally non-susceptible to smoking combustible cigarettes, and participants who were e-cigarette users and were attitudinally non-susceptible to smoking combustible cigarettes.

#### 2.4.2. Exclusion Criteria

Dental students not willing to participate, participants who were combustible cigarette users, participants who were e-cigarette users along with combustible cigarette smoking, and participants experiencing mental health issues or under medication for their health issues.

### 2.5. Data Collection

The data were collected through a structured personal interview from the dental students. Participants in the study were told that no identifiers or personal information would be gathered and that their privacy and confidentiality would be fully maintained. The study subjects provided written, informed consent.

### 2.6. Data Collection Tool

A structured, closed-ended questionnaire that was developed after referring to surveys from previous research that has been published in the literature was used to gather data from the study participants [36,38]. The questionnaire comprised of study participants’ demographic details, their susceptibility to future smoking, smoking habits among family and friends, and details of e-cigarettes among the e-cigarette users.

Study participants meeting the eligibility criteria were assessed for susceptibility to future smoking with 2 items:

“*If one of your friends offered you a cigarette, would you try it?*” and

“*Do you think you will smoke a cigarette sometime in the next year?*”

Responses included “definitely yes”, “probably yes”, “probably no”, and “definitely no”. Those who responded “definitely no” to both measures are considered non-susceptible nonsmokers (NSNSs), whereas those who cannot rule out smoking are defined as susceptible [39,48,51,52,53].

### 2.7. Study Procedure

The study participants were divided into two groups.

Group A comprised participants who were e-cigarette users and were attitudinally non-susceptible to combustible cigarette smoking.

Group B comprised participants who never smoke e-cigarette or combustible cigarettes and are non-susceptible to combustible cigarette smoking. 

**Figure 1:**
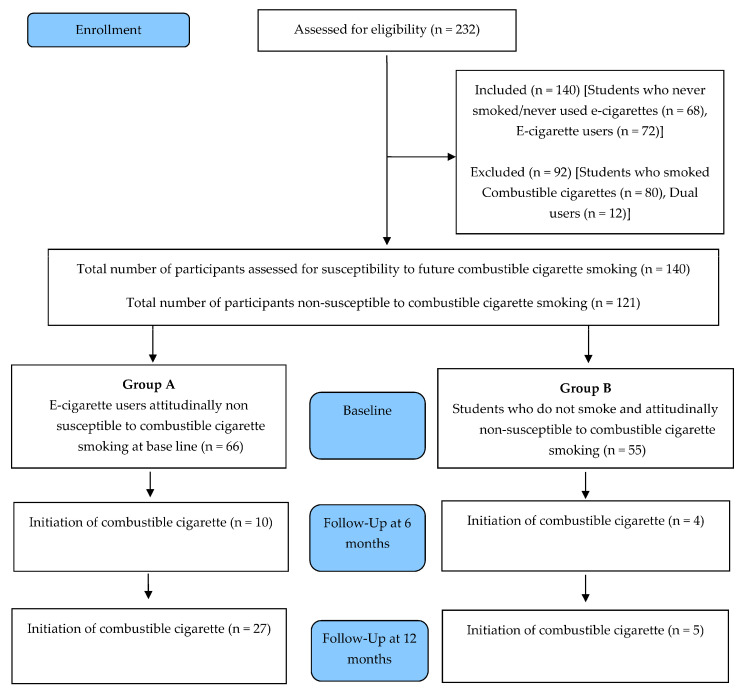
Flowchart of the study design.

Data were collected at baseline and the same groups were followed up 6 months and at the end of 1 year, to assess if there was the initiation of combustible cigarette usage among the two groups [Figure 1].

### 2.8. Data Analysis

Data gathered from the questionnaire were exported into SPSS Statistical Software version 28 (IBM Corporation, Armonk, NY, USA).

Data cleaning was carried out before transferring to SPSS Statistical Software for analysis. Demographic data were summarized as proportions and frequencies. The chi-squared test was used to assess associations between the categorical variables. [Cigarette smoking with age, gender, parental education, parental cigarette smoking, and cigarette smoking among friends]. All these independent parameters significantly based on the chi-squared test were taken forward for binomial regression analysis. A *p* value < 0.05 was considered as statistically significant.

## 3. Results

In the present study, 232 students were approached and requested to participate, amongst which 92 students were excluded as they were either smoking combustible cigarette or were dual users.

A total of 140 students were included, which comprised 68 students who were never users (who never smoked combustible cigarettes or never used e-cigarettes) and 72 students who were e-cigarette users (Table 1).

The 140 participants were further assessed for susceptibility to future smoking. The first statement was “if one of your friends offered you a cigarette, would you try it?”, for which 121 (86.43%) responded with “Definitely No” and 19 (13.57%) “Probably No”. The second statement was “do you think you will smoke combustible cigarette in the next year?”, for which all 140 (100%) responded with “definitely no”.

Following this, 66 (54.6%) were categorized as e-cigarette users attitudinally non-susceptible to combustible cigarette smoking and 55 (45.4%) were categorized as never users attitudinally non-susceptible to combustible cigarette smoking (Table 2).

In the present study, a total of 121 (100%) study participants were assessed at baseline. The mean age of the study participants was 22.80 ± 0.98 years old. A total of 13 (10.7%) participants were 21 years old, 31 (22.6%) were 22 years old, 45 (37.2%) were 23 years old, 31 (25.6%) were 24 years old, and 1 (0.8%) was 25 years old.

A total of 117 (96.7%) were male participants and 4 (3.3%) were female. Parental education of the study participants displayed that fathers’ education for 58 (47.8%) had a Bachelor’s/Master’s degree, 30 (25.0%) had a high-school education, 17 (14.0%) had an intermediate school education, and 16 (13.2%) were illiterate. Mothers’ education displayed that 52 (42.9%) had a Bachelor’s/Master’s degree, 53 (43.8%) had a high-school education, 07 (5.8%) had an intermediate school education, and 09 (7.5%) were illiterate.

Family income of the study participants displayed that 46 (38.0%) had an income less than SAR 10,000, 36 (29.7%) had an income between SAR 10,000–19,000, and 39 (32.3%) had an income of SAR 20,000 and above.

Details of the smoking habits among the study participants’ family and friends were assessed, where 59 (48.8%) mentioned parental cigarette smoking and 77 (63.6%) mentioned smoking among their friends (Table 3).

The initiation of combustible cigarette smoking at 6-month and 12-month intervals among the groups showed that, at 6 months, of the 66 e-cigarette users, 10 (15.1%) students had tried combustible cigarette smoking and at the end of 1 year, 27 (40.9%) students reported to have used a combustible cigarette at some point during the follow up. At the same time, 4 (7.2%) students at 6 months and 5 (9.09%) at 12 months, who had reported absolutely no form of cigarette usage at baseline, reported to have tried combustible cigarettes during the follow up (Table 4).

Chi-squared analysis of independent variables revealed e-cigarette use to be significantly associated with fathers’ education level (*p* = 0.00), parental cigarette smoking status (*p* = 0.00), cigarette smoking among friends (*p* = 0.00), and family income (*p* = 0.00). The e-cigarette users are more likely to believe it to be healthier (*p* = 0.00) than combustible smoking (Table 5).

Binomial regression analysis of the outcome at the end of the one-year follow-up when analyzed with independent variables revealed a significant influence of e-cigarette use on taking up combustible cigarette use [Relative Risk (RR): 9.395; 95% CI: 3.03–29.04]. Fathers’ education level negatively (the higher the education, the less likely their wards were to take up combustible cigarette smoking) influences the uptake of combustible cigarettes [RR: 0.180; 95% CI: 0.054–0.604]; likewise, a higher family income (SAR > 20,000) was more likely to negatively influence the taking up of combustible cigarette smoking [RR: 0.026; 95% CI: 0.00–0.20]. Parental cigarette smoking was seen to positively influence the initiation of combustible cigarette use [RR: 2.667; 95% CI: 1.24–5.73] (Table 6)].

## 4. Discussion

The usage of e-cigarettes among youths across the globe has been increasing [54,55]. The younger population has shown a higher tendency to use and experiment with e-cigarettes, which is seen in both combustible cigarette users and non-users [53,56,57,58].

Saudi Arabia permits the sale of e-cigarettes and does not impose any restrictions on e-cigarette sponsorship, promotion, or advertising [59]. According to Saudi Arabia’s smoking statistics, 12.1% of people currently smoke tobacco, with a prevalence of 23.7% for men and 1.5% for women. Moreover, the data reveal that 60.9% of them began smoking before the age of eighteen years old, and 29.7% of them did so before the age of fifteen years old [60]. In Saudi Arabia, the percentage of medical students who used e-cigarettes ranged from 10.6% to 27.7% [28].

Dental professionals are essential for the development and implementation of smoking prevention and cessation initiatives for their patients [61,62]. Dental practitioners can effectively assist patients in quitting smoking and are in a good position to detect early indicators of tobacco use in the oral cavity [63,64,65,66]. Additionally, the World Health Organization places a strong emphasis on teaching and training oral health professionals in the early identification and detection of tobacco’s harmful effects, as well as the planning of early intervention and treatment for those effects [67]. Nonetheless, medical personnel who smoke might not be proficient and successful in helping people quit smoking [68,69]. Dentists’ attitudes and understanding of e-cigarettes may influence how involved they are in smoking cessation programs. 

Dental students in Malaysia had an inadequate understanding of electronic cigarettes, according to Sobri M et al. [70]. Of the study participants, 62.1% had an inadequate awareness about e-cigarettes, and 56% thought they were less harmful. In a study on health professional students’ understanding of e-cigarettes in Saudi Arabia, Alsanea S et al. [71] found that participants had misconceptions, considered e-cigarettes as a tool for quitting smoking, and knew hardly anything about the harmful effects of e-cigarettes. According to a research article by Kurdi R et al. [72], university students in Qatar who use e-cigarettes felt that they are safer than traditional cigarettes, and most of them thought that using e-cigarettes would help them avoid using conventional cigarettes. Another investigation by Wamamili B et al. [73] among New Zealand university students found that 76.1% of the subjects thought e-cigarettes were safer than conventional cigarettes. In the present study, 52.0% of participants thought that electronic cigarettes were healthier than combustible cigarettes.

In the present study, the mean age of the study participants was 22.80 ± 0.98 years old. Multiple similar studies were conducted on participants whose mean ages were 17.1 years [44], 14.1 years [36], 14.7 years [37], 16–26 years [38], and 14.3 years old [41]. These results indicate that young people are more likely to start smoking while they are younger. Since there is no research on dental students in the literature, we are contrasting our results with those of studies on a younger group.

In the present study, the susceptibility of the study participants toward future combustible cigarette initiation was assessed using validated measures [44,51,52,53,74]. Only the study participants who responded “definitely no” to the questions assessing their intention to initiate combustible cigarettes in the future were enrolled. The current study revealed that those who had previously used e-cigarettes had a higher risk of initiating combustible cigarettes [RR: 9.395; 95% CI: 3.03–29.04]. Compared to earlier research published in the literature, these results were higher. According to JL Barrington-Trimis et al. [44], the OR for e-cigarette users was 1.75 (95% CI: 1.10–2.77). Wills TA et al. [37] also reported similar findings, indicating that e-cigarette users had 2.87 (95% CI: 2.03–4.05) times the OR of initiating to use combustible cigarettes. According to Leventhal AM et al. [36], e-cigarette users had a 4.27 [95% CI: 3.19–5.61] increased risk of initiating to use combustible cigarettes at two follow-up periods compared to non-users. In another investigation, Primack BA et al. [38] found an independent correlation between e-cigarette use and the start of combustible smoking (OR = 8.3; 95% CI: 1.2–58.6). In another study on secondary-school students, Hammond D. et al. [20] found that baseline e-cigarette use was linked to initiating the use of combustible cigarettes at the end of the year, with an OR of 2.12 [95% CI: 1.68–2.66]. A longitudinal cohort study by Watkins SL et al. [41] among children aged 12 to 17 years old revealed that e-cigarette users had advanced to smoking combustible cigarettes (OR = 1.87; 95% CI: 1.15–3.05.). In another study, Conner M et al. [42] examined school-age children aged 13 to 14 years old and discovered a high correlation between baseline e-cigarette use and the future commencement of combustible cigarette smoking, with an OR of 5.38 [95% CI: 4.02 to 7.22]. 

According to a similar study by Friedman AS et al. [47], e-cigarette use was positively correlated with young people’s decision to start smoking combustible cigarettes, with an adjusted OR of 6.75 [95% CI: 3.93–11.57; *p* = 0.001]. Another study by Staff, J. et al. [75] found that study participants who started using e-cigarettes by the age of 14 years old were more likely to start smoking cigarettes by the age of 17 years old, with an OR = 5.25 [95% CI = 3.28–8.38], compared to non-users. Additionally, according to Xu, S. et al. [76], using e-cigarettes increased the likelihood of starting to smoke combustible cigarettes subsequently (OR = 3.42; 95% CI = 1.99, 5.93). Our results, however, are not in line with those of a study published by Shahab L et al. [77], which revealed that e-cigarette users had lower odds of starting to smoke combustible cigarettes (OR: 0.76; 95% CI: 0.62 to 0.93) than non-users did. Our research and published studies strongly suggest that the use of e-cigarettes by individuals may act as an initiator or enabler for the initiation of combustible smoking. This is because the flavor of and nicotine in e-cigarettes have the potential to become addictive, which may lead to users’ intention to smoke or to initiate the use of combustible cigarettes [48]. It is important to recognize that using e-cigarettes increases the risk of starting to smoke combustible cigarettes. 

In the current study, fathers’ educational attainment had a negative influence on their wards’ likelihood of starting to smoke combustible cigarettes (more education = lower likelihood of combustible cigarette smoking [RR: 0.180; 95% CI: 0.054–0.604]. These results align with those of a study carried out by Alves, J. et al. [78]. Teenagers who have smoking parents are more likely to start smoking themselves. According to a meta-analysis published by Leonardi-Bee J et al. [79], boys who had parents with lower levels of education had greater odds of smoking (OR: 1.57; 95% CI: 1.01–1.43). Zaloudikova I et al. [80] found that children who have a better parental education and status are less likely to experiment with smoking cigarettes.

Research has indicated that dental students who participate in tobacco prevention education initiatives at their universities have witnessed a decrease in social nicotine dependence [81]. Since Saudi Arabia is an Islamic country that views smoking as a sin, it is important to address the issue of smoking among students pursuing health professions [82]. It is necessary to create educational initiatives in addition to professional counseling and pharmacological therapies, taking into account the health issues facing the next generation of doctors and their position in society as facilitators of smoking cessation interventions. Research by Maharani, D.A. et al. [83], and Priya, H. et al. [84] has brought attention to the necessity of creating dentistry curricula that encourage professional accountability for smoking cessation programs. This can improve dental students’ knowledge and self-assurance when participating in patient-centered tobacco cessation therapies [83,84]. The reasons provided by dental students for not participating in smoking cessation programs in dental schools included: using tobacco products themselves; lacking confidence; thinking that dental clinics are not appropriate places for counseling; and feeling unqualified to assist patients in quitting smoking [84,85].

In Riyadh, Saudi Arabia, dental students’ opinions toward smoking cessation counseling were documented by Alhussain A.A. et al. [86], where 72.9% of study participants said they would be interested in receiving training to help their patients quit smoking. However, 67% of the study participants considered a lack of training on smoking cessation and counseling as a barrier to helping patients quit smoking. Several industrialized nations, like the United States and Canada, prioritize knowledge-based tobacco teaching primarily in their dentistry and medical curricula [87,88]. Since the dental educational system lacks formal instruction, there is also a great deal of heterogeneity in the attitudes of dental students toward smoking cessation programs [89].

Incorporating smoking cessation counseling and intervention training programs into oral health education should be a priority, as should policy development and curricular modifications. Additionally, dental schools ought to place a stronger emphasis on education and skill development by actively involving dental students in patient interventions for smoking prevention and cessation [90,91].

A few of the study’s limitations were the study participants, the majority of whom were male students; this finding may have been caused by the female students’ tendency toward social desirability bias. The study’s second limitation was that all of the participants were from the same province and institution. The third limitation involved comparing the results of these studies with the research on younger populations, given the lack of studies involving dental students.

### Implications of the Study

This study’s results show that using e-cigarettes increases the likelihood of starting to use combustible cigarettes among health professional students who are not predisposed to smoking. Strict regulations should be created to restrict smoking among health university students in recognition of the study’s findings. Dental professionals should be inspired to be role models for both society and their patients, as they are crucial in the creation and implementation of smoking prevention and cessation programs for their patients. Since this is the first study of its kind to report on dental students in the literature, the findings may be used to inform future health professionals about the harmful consequences of e-cigarettes. Subsequent research endeavors ought to encompass a greater number of participants over an extended study period at various institutions and provinces. It is important to plan future research to find the most effective strategies for helping health professionals stop smoking combustible cigarettes and e-cigarettes.

## 5. Conclusions

According to the current study, people who have previously used e-cigarettes are more likely to initiate smoking combustible cigarettes. In Saudi Arabia, the sales and accessibility of e-cigarettes have led to a sharp rise in the use of these products among teenagers and young people. The observations show that parental cigarette smoking influences children’s decision to start smoking combustible cigarettes. A total of 52.0% of the study’s participants believed that e-cigarettes were healthier than combustible cigarettes. Educational institutions should implement stringent policies and regulations to prevent the use of products among health professionals. Educational campaigns that aim to inform youth and families about the risks of e-cigarette usage should create public awareness about the safety of these e-cigarettes. Tobacco control laws and prevention initiatives, in addition to e-cigarette regulations, are required to lower the number of young people who will smoke combustible cigarettes in the future. We should plan future research to identify the most effective strategies for helping young people stop using e-cigarettes. Dental care providers should receive sufficient training and support in order to effectively screen and counsel their patients about e-cigarette usage.

## Figures and Tables

**Table 1:** Distribution of study participants based on their smoking habits.

Study Parameters		Frequency	Percentage
Current use of tobacco	Yes	164	70.7%
	No	68	29.3%
	Total	232	100%
Type of cigarette usage	Combustible cigarette	80	48.8%
	Electronic cigarette	72	43.9%
	Both	12	7.3%
	Total	164	100%

**Table 2:** Distribution of study participants based on their susceptibility to future smoking of combustible cigarettes.

Statements	Responses	Frequency	Percentage
If one of your friends offered you a cigarette, would you try it?	Definitely Yes	0	0%
	Probably Yes	0	0%
	Definitely No	121	86.43%
	Probably No	19	13.57%
Do you think you will smoke combustible cigarette in the next year?	Definitely Yes	0	0%
	Probably Yes	0	0%
	Definitely No	140	100%
	Probably No	0	0%

**Table 3:** Distribution of study participants based on demographic details.

Study Parameters		Frequency	Percentage
Age group wise distribution	21	13	10.7%
	22	31	25.6%
	23	45	37.2%
	24	31	25.6%
	25	1	0.8%
	Mean Age	22.80 ± 0.98	
Biological sex	Male	4	3.3%
	Female	117	96.7%
Father’s education	Illiterate	16	13.2%
	Intermediate School	17	14.0%
	High School	30	25.0%
	Bachelor’s/Master’s Degrees/Above	58	47.8%
Mother’s education	Illiterate	09	7.5%
	Intermediate School	07	5.8%
	High School	53	43.8%
	Bachelor’s/Master’s Degrees/Above	52	42.9%
Family income	Less than SAR 10,000	46	38.0%
	SAR 10,000–19,000	36	29.7%
	SAR 20,000 and Above	39	32.3%
Parental cigarette smoking	Yes	59	48.8%
	No	62	51.2%
Smoking among friends	Yes	77	63.6%
	No	44	36.4%
Total		121	100%

**Table 4:** Distribution of study participants based on the initiation of combustible cigarettes at follow up.

Study Parameters	At Baseline		At 6 Months		At 12 Months	
	Frequency	Percentage	Frequency	Percentage	Frequency	Percentage
Usage of combustible cigarettes among e-cigarette users	Nil	Nil	10	15.1%	27	40.9%
Usage of combustible cigarettes among never-users	Nil	Nil	4	7.2%	5	9.09%

**Table 5:** Association of independent variables with the participants’ smoking habits.

Independent Variable	Dependent Variable	Chi-Square Test	df	**Sign**
Mother’s Education	Do you smoke?	0.96	3	0.81
Father’s Education		34.68	3	0.00 *
Parental Cigarette Smoking		13.44	1	0.00 *
Parental Cigarette Smoking	What do you smoke?	13.44	1	0.00 *
Cigarette Smoking among Friends		16.94	1	0.00 *
Family Income		37.74	2	0.00 *
Electronic Cigarette is Healthier than Regular Combustible Cigarettes		74.61	1	0.00 *

* = statistically significant.

**Table 6:** Influence of independent variables on the participants’ smoking habits.

Variable	df	Sign	Exp (B)	95% Confidence Interval of RR	
				Lower	Upper
E-cigarette ref: non-user	1	0.000	9.395	3.039	29.045
Family income (SAR 20,000 and above) Family Income (SAR 10,000–19,000) Ref: SAR < 10,000	1	0.012	0.286	0.108	0.758
	1	0.001	0.026	0.003	0.208
Father education: Bachelor’s/Master’s degrees	1	0.005	0.180	0.054	0.604
Father education: high school	1	0.487	0.651	0.195	2.179
Father education: intermediate school ref: illiterate	1	0.356	0.511	0.123	2.122
Parental cigarette smoking ref: non-users	1	0.012	2.667	1.241	5.730
Omnibus Test of Model Coeiffcient		*p* = 0.000	Nagelkerke R Square	0.307	

## Data Availability

Data are contained within the article.
